# Environmental and Genetic Contributors to Salivary Testosterone Levels in Infants

**DOI:** 10.3389/fendo.2014.00187

**Published:** 2014-10-30

**Authors:** Kai Xia, Yang Yu, Mihye Ahn, Hongtu Zhu, Fei Zou, John H. Gilmore, Rebecca C. Knickmeyer

**Affiliations:** ^1^Department of Psychiatry, University of North Carolina at Chapel Hill, Chapel Hill, NC, USA; ^2^Department of Statistics and Operations Research, University of North Carolina at Chapel Hill, Chapel Hill, NC, USA; ^3^Department of Biostatistics, University of North Carolina at Chapel Hill, Chapel Hill, NC, USA

**Keywords:** testosterone, twins, infancy, APGAR, *LHCGR*, hypothalamic–pituitary–gonadal axis, minipuberty, neonate

## Abstract

Transient activation of the hypothalamic–pituitary–gonadal axis in early infancy plays an important role in male genital development and sexual differentiation of the brain, but factors contributing to individual variation in testosterone levels during this period are poorly understood. We measured salivary testosterone levels in 222 infants (119 males, 103 females, 108 singletons, 114 twins) between 2.70 and 4.80 months of age. We tested 16 major demographic and medical history variables for effects on inter-individual variation in salivary testosterone. Using the subset of twins, we estimated genetic and environmental contributions to salivary testosterone levels. Finally, we tested single nucleotide polymorphisms (SNPs) within ±5 kb of genes involved in testosterone synthesis, transport, signaling, and metabolism for associations with salivary testosterone using univariate tests and random forest (RF) analysis. We report an association between 5 min APGAR scores and salivary testosterone levels in males. Twin modeling indicated that individual variability in testosterone levels was primarily explained by environmental factors. Regarding genetic variation, univariate tests did not reveal any variants significantly associated with salivary testosterone after adjusting for false discovery rate. The top hit in males was rs10923844, an SNP of unknown function located downstream of *HSD3B1* and *HSD3B2*. The top hits in females were two SNPs located upstream of *ESR1* (rs3407085 and rs2295190). RF analysis, which reflects joint and conditional effects of multiple variants, indicated that genes involved in regulation of reproductive function, particularly *LHCGR*, are related to salivary testosterone levels in male infants, as are genes involved in cholesterol production, transport, and removal, while genes involved in estrogen signaling are related to salivary testosterone levels in female infants.

## Introduction

Transient activation of the hypothalamic–pituitary–gonadal (HPG) axis in the early post-natal period results in elevated levels of gonadotropins and testosterone in human males, a phenomenon known as the “minipuberty” or “neonatal surge” (1–5). The consequences of this activation are not fully understood, but it likely plays an important role in genital development (6–9) and has been linked to future fertility (10). There is also an increasing body of evidence that this is a critical period for the development of sexually dimorphic behavior and psychopathology (11–16). Understanding the causes of individual variation in testosterone levels during the minipuberty is thus of considerable theoretical and clinical interest.

Levels of pubertal and post-pubertal testosterone in males are determined, in part, genetically, with heritability estimates around 40–70% (17–23) for both salivary and plasma measures. Studies in females are less consistent with some reporting minimal heritable variation (23) and others reporting heritabilities similar to those seen in males (17, 20, 22). Recently, genome-wide association studies (GWAS) have been used to identify single nucleotide polymorphisms (SNPs) associated with serum levels of testosterone in adults. Ohlsson et al. (24) identified two loci that met genome-wide significance for serum testosterone levels in men of European ancestry, one in the gene for sex hormone-binding globulin (*SHBG*, lead SNPs rs12150660 and rs6258) and one near *family with sequence similarity 9, member B* (*FAM9B*) on Xp22 (lead SNP rs5934505). Jin et al. (25) confirmed both loci in an independent study, although the lead SNP in the *SHBG* locus differed (rs727428). They also reported a new hit at rs10822184, which is located in *receptor accessory protein 3* (*REEP3*). Chen et al. (26) reported a significant association between rs2075230 in the *SHBG* locus and serum testosterone in a sample of Chinese men. A GWAS of 1600 post-menopausal women failed to find any genome-wide significant associations with testosterone (27).

There is evidence to suggest that distinct genetic mechanisms influence testosterone levels across developmental time (17), so it is theoretically possible that the relative importance of genetic factors and the specific variants involved may be different in the minipuberty. Indeed, a study of salivary testosterone in twins between 4 and 8 months of age reported that variation was completely explained by common and unique environmental factors (28). This result is extremely intriguing, but the age range studied is slightly beyond the minipuberty as classically defined. Most studies suggest that the post-natal surge in testosterone peaks in males between 1 and 3 months of age and declines significantly by 6 months of age (4, 5, 29, 30). The gonads are the main source of testosterone in males during the minipuberty, but after 6 months the adrenal becomes the primary source of testosterone (31) as a consequence of the regression and degeneration of neonatal Leydig cells (32). Complementary studies at earlier ages are therefore needed as are studies looking at the impact of specific environmental factors.

The current study takes advantage of an ongoing GWAS of infant brain development being carried out at the University of North Carolina (UNC) at Chapel Hill. The study includes both twins and singletons. A subset of participating children donated saliva samples for the assessment of salivary testosterone around 3 months of age [see Ref. (33)]. We used this sample to estimate genetic and environmental contributions to salivary testosterone levels during the minipuberty. We also examined the impact of 16 major demographic and medical history variables on inter-individual variation in salivary testosterone. In addition, we tested SNPs within ±5 kb of genes involved in testosterone synthesis, transport, signaling, and metabolism for their relationship with salivary testosterone. We also tested SNPs in *REEP3* and the Xp22 loci identified in GWAS studies of serum testosterone in adult males to see if these were predictive in infants. Finally, to address the issue of potentially small effect sizes for individual SNPs, as well as non-additive effects, we used random forest (RF) methods to identify combinations of SNPs contributing to variation in salivary testosterone in infancy.

## Materials and Methods

### Subjects

Two-hundred twenty-two infants (119 males, 103 females) between 2.70 and 4.80 months of age (mean age 3.34 months post-date of birth ± 0.36) with high quality genetic information and saliva samples suitable for hormone assay are included in this analysis. The sample includes 108 singletons and 114 twins (27 same-sex DZ pairs, 27 same-sex MZ pairs, 2 opposite-sex DZ pairs, and 2 unpaired twins). All children were participating in prospective longitudinal studies of early brain development for which the senior author is a co-investigator. Mothers were recruited during the second trimester of pregnancy from the outpatient obstetrics and gynecology clinics at UNC hospitals. Exclusion criteria at enrollment were the presence of abnormalities on fetal ultrasound or major medical illness in the mother. Demographic variables (maternal age, paternal age, maternal education, paternal education, maternal ethnicity, paternal ethnicity, maternal psychiatric history, paternal psychiatric history, and total household income) were collected via maternal report at the time of enrollment (see Supplementary Material). For the purpose of the current study, maternal psychiatric history and paternal psychiatric history were treated as binary variables. Individuals were counted as positive for psychiatric history if they had received any psychiatric diagnosis. Medical history variables (birth weight, gestational age at birth, 5 min APGAR scores, stay in neonatal intensive care unit over 24 h, gestation number, and delivery method) were collected from maternity and pediatric medical records shortly after birth (see Supplementary Material). Maternal smoking during pregnancy was collected via maternal report at two timepoints during pregnancy and shortly after birth. Demographic and medical history data are summarized in Table 1. Experiments were undertaken with the understanding and written consent of each subject’s mother or father, with the approval of the Institutional Review Board of the UNC School of Medicine.

**Table 1:** **Demographic and medical history information**.

	Male (*N* = 119)	Female (*N* = 103)
**CONTINUOUS VARIABLES Mean (SD)**		
Age since DOB^a^ (months)	3.32 (0.36)	3.36 (0.37)
Gestational age at birth (weeks)	37.3 (3.0)	37.7 (2.3)
Birth weight (g)	2886 (816)	2910 (563)
5-Min APGAR Score	8.7 (0.7)	8.6 (0.7)
Maternal age (years)	29.7 (6.1)	30.3 (5.4)
Paternal age (years)	31.5 (5.9)	32.5 (6.1)
Maternal education (years)	15.7 (3.3)	15.7 (3.0)
Paternal education (years)	15.7 (3.1)	15.0 (3.0)
**CATEGORICAL VARIABLES NO. (%)**		
Gestation number	Twin 65 (55%)	Twin 49 (48%)
	Singleton 54 (45%)	Singleton 54 (52%)
NICU stay >24 h	No 98 (82%)	No 86 (83%)
	Yes 21 (18%)	Yes 17 (17%)
Caesarian section	No 60 (50%)	No 56 (54%)
	Yes 59 (50%)	Yes 47 (46%)
Maternal ethnicity	White 89 (75%)	White 82 (80%)
	Black 24 (20%)	Black 20 (19%)
	Asian 4 (3%)	Asian 1 (1%)
	Other 2 (1%)	
Paternal ethnicity	White 86 (72%)	White 81 (79%)
	Black 26 (22%)	Black 19 (18%)
	Asian 7 (6%)	Asian 3 (3%)
Maternal psychiatric history	No 93 (78%)	No 81 (79%)
	Yes 26 (22%)	Yes 22 (21%)
Paternal psychiatric history	No 103 (87%)	No 94 (91%)
	Yes 16 (13%)	Yes 9 (9%)
Income^b^	High (29%)	High (33%)
	Middle (39%)	Middle (37%)
	Low (30%)	Low (28%)
	Missing (2%)	Missing (2%)
Maternal smoking	No 107 (90%)	No 93 (90%)
	Yes 12 (10%)	Yes 10 (10%)

^a^DOB = date of birth,

*^b^low income: at or below 200% of federal poverty level (FPL), middle income: between 200 and 400% of FPL, high income: above 400% of FPL*.

### Salivary assays

Saliva samples were collected during visits to participants’ homes. Visits were scheduled for 9:00 a.m. Mean ± SD for collection start time was 9:14 a.m. ± 16 min. Parents were advised not to feed their children for at least 15 min prior to collection. One milliliter of passive drool was collected from each participant using a suction catheter (Centurion Healthcare Products, Howell, MO, USA). Mean ± SD for duration of collection was 4 ± 2 min. All samples were frozen within 5 h and stored in a −80 °C freezer. Mean ± SD for time until storage was 60 ± 31 min.

Salivary testosterone levels were measured by enzyme immunoassay using a commercially available kit (Salimetrics, State College, PA, USA). The intra-assay precision for samples with low testosterone levels (mean 18.12 pg/ml) is 6.7%; for high testosterone levels (mean 188.83 pg/ml) it is 2.5%. Inter-assay precision for samples with low testosterone levels (mean 19.6 pg/ml) is 14.05%; for high testosterone levels (mean 199.08 pg/ml) it is 5.6%. Percent recovery for this assay varies from 92 to 111.4%. The minimal concentration of testosterone that can be distinguished from 0 is <1.0 pg/ml. Only one sample had a concentration below the detection limit. This sample was coded as 0 pg/ml in subsequent analyses. We also evaluated all samples for blood contamination using the Salimetrics Salivary Blood Contamination Enzyme Immunoassay kit, which quantitatively measures transferrin, a large protein, which is present in abundance in blood, but that is normally present in only trace amounts in saliva. Intra-assay precision for samples with high (3.88 mg/dl) transferrin levels is 10.2%, for samples with low (0.42 mg/dl) transferrin levels it is 4.9%. Inter-assay precision is 7.1% for low (1.02 mg/dl) and 7.2% for high (4.93 mg/dl) transferrin levels. Percent recovery varies from 91.9 to 101.5%. The minimal concentration of transferrin that can be distinguished from zero is 0.08 mg/dl.

### Genotyping

**Figure 1:**
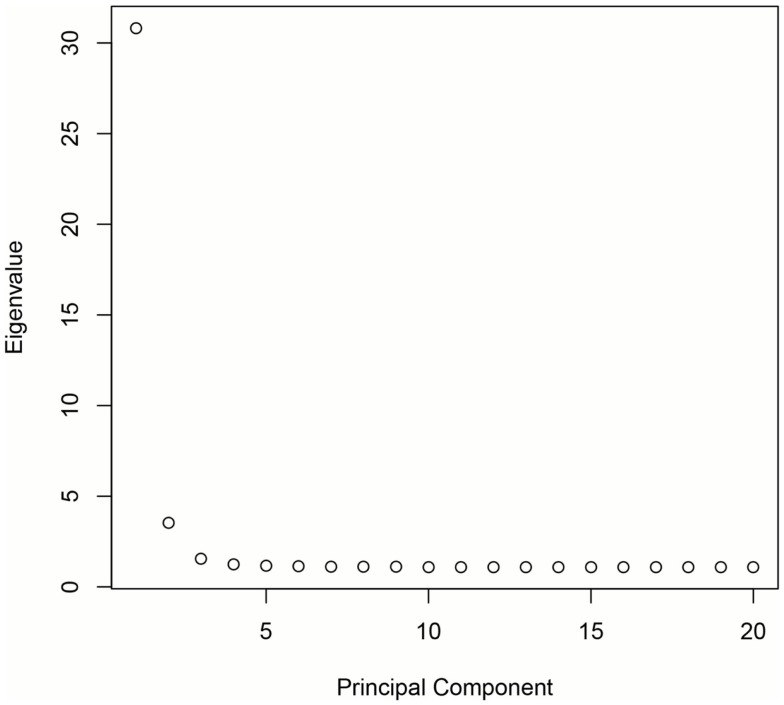
**Scree plot demonstrating that the majority of genetic variation is explained by the first three PCs**.

**Figure 2:**
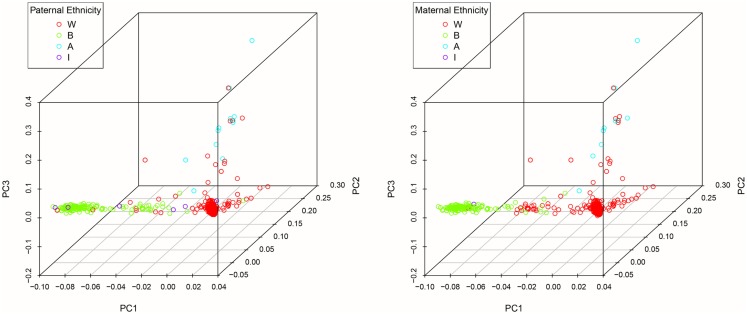
**3D scatter plots of the first three PCs color-coded by maternal and paternal reported ethnicity**.

DNA was extracted from buccal cells using standard methods as described in the Puregene^®^ DNA Purification Kit (Gentra Systems) or using phenol/chloroform. After extraction, samples were stored in a −80 °C freezer until analysis. Genotyping was carried out at the Bionomics Research and Technology Center at Rutgers (Piscataway, NJ, USA) using Affymetrix Axiom Genome-Wide LAT and Exome arrays. Samples were randomized across 96-well plates. Each plate contained a common control sample. Genotype calling was performed with the Affymetrix Genotyping Console. Rigorous quality control procedures were carried out prior to analysis. In brief, we excluded samples with low DishQC (<0.82 for LAT array and <0.79 for Exome array), low call rates (<95%), outliers for homozygosity, sex, or zygosity from genotypes inconsistent with reported phenotypes, ancestry outliers, excessive relatedness, and unexpected relatedness. We also removed individual SNPs that deviated from Hardy–Weinberg equilibrium (*P*_HWE_ < 1 × 10^−8^), had low call rate (<95%), high deviation of allele frequency from 1000G EUR/AFR founders, and that did not match 1000G EUR/AFR founders. Population stratification was assessed using principal component analysis (PCA) (34, 35). Population stratification is the presence of systematic differences in allele frequencies between subpopulations with different genetic ancestry and can lead to spurious results in genetic association studies. We first pruned the genotyped SNPs using the pruning tool in PLINK (36). We then created a subset of our total GWAS sample without related subjects by randomly selecting one individual from twin and sibling pairs (all non-twins/non-sibs were also included). Smartpca in EIGENSOFT was applied to compute the principal components (PCs or eigenvectors). The first three PCs explain 6.8% of the variability in genetic variables. See Figures 1 and 2 for a scree plot and 3D scatter plots of the first three PCs color-coded by maternal and paternal reported ethnicity. Strong clustering of subjects with similar reported ethnicity confirms that the first three PCs index genetic ancestry. Imputation was then performed with MaCH-Admix using 1000G reference panel (phase1_release_v3.20101123) followed by post-imputation quality control based on imputation quality score.

For the current analysis, we extracted all genotyped SNPs within ±5 kb of genes involved in testosterone synthesis, transport, signaling, and metabolism (see Table 2). We also included genotyped SNPs in/near *REEP3* and the Xp22 loci flagged in GWAS studies of serum testosterone in adult males. Three of the top GWAS hits in *SHBG* were not genotyped by either array, but were imputed with good quality (rs727428, rs12150660, and rs2075230). The imputed SNPs were used in the current analysis. rs6258 was directly genotyped, but was excluded due to low minor-allele frequency (MAF). Top hits in *REEP3* and the Xp22 loci were also imputed (rs10822184 and rs5934505). Additional imputed SNPs near the Xp22 loci were also included due to the low number of genotyped SNPs in this region. SNPs with MAF <0.05 were not included in our analyses. In total, 512 SNPs were examined in males and 473 SNPs were examined in females.

**Table 2:** **Genes probed in the current analysis**.

Gene symbol	Gene name	General category
*CYP11A*	Cytochrome P450, family 11, subfamily A, polypeptide 1	Synthesis and metabolism
*CYP11B1*	Cytochrome P450, family 11, subfamily B, polypeptide 1	Synthesis and metabolism
*CYP17A1*	Cytochrome P450, family 17, subfamily A, polypeptide 1	Synthesis and metabolism
*CYP19A1*	Cytochrome P450, family 19, subfamily A, polypeptide 1 (Aromatase)	Synthesis and metabolism
*CYP1B1*	Cytochrome P450, family 1, subfamily B, polypeptide 1	Synthesis and metabolism
*CYP21A2*	Cytochrome P450, family 21, subfamily A, polypeptide 2	Synthesis and metabolism
*CYP3A4*	Cytochrome P450, family 3, subfamily A, polypeptide 4	Synthesis and metabolism
*CYP3A43*	Cytochrome P450, family 3, subfamily A, polypeptide 43	Synthesis and metabolism
*CYP3A5*	Cytochrome P450, family 3, subfamily A, polypeptide 5	Synthesis and metabolism
*CYP3A7*	Cytochrome P450, family 3, subfamily A, polypeptide 7	Synthesis and metabolism
*CYP7A1*	Cytochrome P450, family 7, subfamily A, polypeptide 1	Synthesis and metabolism
*DHCR7*	7-dehydrocholesterol reductase	Synthesis and metabolism
*HSD17B1*	Hydroxysteroid (17-beta) dehydrogenase 1	Synthesis and metabolism
*HSD17B2*	Hydroxysteroid (17-beta) dehydrogenase 2	Synthesis and metabolism
*HSD17B3*	Hydroxysteroid (17-beta) dehydrogenase 3	Synthesis and metabolism
*HSD17B6*	Hydroxysteroid (17-beta) dehydrogenase 6	Synthesis and metabolism
*HSD17B7*	Hydroxysteroid (17-beta) dehydrogenase 7	Synthesis and metabolism
*HSD17B8*	Hydroxysteroid (17-beta) dehydrogenase 8	Synthesis and metabolism
*HSD3B1*	Hydroxy-delta-5-steroid dehydrogenase, 3 beta- and steroid delta-isomerase 1	Synthesis and metabolism
*HSD3B2*	Hydroxy-delta-5-steroid dehydrogenase, 3 beta- and steroid delta-isomerase 2	Synthesis and metabolism
*POR*	P450 (cytochrome) oxidoreductase	Synthesis and metabolism
*SRD5A1*	Steroid-5-alpha-reductase, alpha polypeptide 2 (3-oxo-5 alpha-steroid delta 4-dehydrogenase alpha 1)	Synthesis and metabolism
*SRD5A2*	Steroid-5-alpha-reductase, alpha polypeptide 2 (3-oxo-5 alpha-steroid delta 4-dehydrogenase alpha 2)	Synthesis and metabolism
*SRD5A3*	Steroid 5 alpha-reductase 3	Synthesis and metabolism
*STAR*	Steroidogenic acute regulatory protein	Synthesis and metabolism
*STS*	Steroid sulfatase (microsomal), isozyme S	Synthesis and metabolism
*SULT2A1*	Sulfotransferase family, cytosolic, 2a, dehydroepiandrosterone (DHEA)-preferring, member 1	Synthesis and metabolism
*SHBG*	Sex hormone-binding globulin	Transport
*TSPO*	Translocator protein (18 kDa)	Transport
*ALB*	Albumin	Transport
*AR*	Androgen receptor	Receptors
*ESR1*	Estrogen receptor alpha	Receptors
*ESR2*	Estrogen receptor beta	Receptors
*CGA*	Glycoprotein hormones, alpha polypeptide	Regulators of reproductive function
*GNRH1*	Gonadotropin-releasing hormone 1 (luteinizing-releasing hormone)	Regulators of reproductive function
*LHB*	Luteinizing hormone beta polypeptide	Regulators of reproductive function
*GNRHR*	Gonadotropin-releasing hormone receptor	Regulators of reproductive function
*LHCGR*	Luteinizing hormone/choriogonadotropin receptor	Regulators of reproductive function
*REEP3*	Receptor accessory protein 3	GWAS hit for serum testosterone
*Xp22 near rs5934505*	Closest gene is family with sequence similarity 9, member B	GWAS hit for serum testosterone

### Environmental association analysis

In order to determine the potential impact of major demographic and medical history variables on inter-individual variation in salivary testosterone, we used a moment-based method to select fixed effects in linear mixed effects models (37). Twins are treated as repeated measures. For fixed effects selection, we applied an adaptive Lasso penalty using the feasible generalized least squares estimator as an initial. In the model, we always include log(10) transferrin and age since DOB as predictors. We focused on selecting a list of predictors including maternal age, paternal age, maternal education, paternal education, maternal ethnicity, paternal ethnicity, maternal psychiatric history, paternal psychiatric history, total household income, maternal smoking during pregnancy, birth weight, gestational age at birth, 5 min APGAR scores, stay in neonatal intensive care unit over 24 h, gestation number, and delivery method. We used the BIC statistic to select the tuning parameter of the adaptive Lasso as in Section 4 of Ahn et al. (37). Before applying our variable selection method, we standardized all covariates and centered the response variable (testosterone). We also applied bootstrap methods 1000 times to assess the stability of our results.

After model selection, we ran a mixed effect model using the selected variables for significance testing and to estimate *r*^2^ values. Mixed effect models were also run including all variables for comparison. Males and females were analyzed separately for this and all subsequent analyses. We separated the sexes because the source of testosterone is primarily gonadal in males and adrenal in females during the minipuberty (31). Studies in adults also suggest that different genetic factors influence testosterone concentrations in men and women (17). Variables were considered significant if they were selected in the Lasso model and survived Bonferroni correction in the mixed effect model including all variables.

### Intraclass correlation coefficient and heritability estimation

The intraclass correlation coefficient (ICCs) and their confidence intervals were estimated separately for MZM (monozygotic male), DZM (dizygotic male), MZF (monozygotic female), and DZF (dizygotic female) using R package called ICC. The heritability was estimated using linear mixed effect model (ACE model) while restricting variance of genetic, shared environment, and random error to be >0. One-tailed *t*-tests were used to test whether the estimates were significantly >0 (*p*-values <0.05 were considered significant). For males, log(10)transferrin, age since DOB, and 5 min APGAR score were included as covariates. For females, log(10)transferrin and age since DOB were included as covariates.

### Univariate genetic association analysis

Males and females were analyzed separately. For males, log(10)transferrin, age since DOB, and 5 min APGAR score were included as covariates. For females, log(10)transferrin and age since DOB were included as covariates. In addition, the first three principle components derived from all genotyped SNPs were included as covariates to control for possible population stratification. Association analysis was performed using mixed effect models with likelihood ratio tests. *p*-values were adjusted by false discovery rate (FDR); *p*-values <0.05 after FDR correction were considered significant.

Univariate genetic association analysis provides a straightforward approach to identifying genetic variants associated with phenotypes of interest, in this case salivary testosterone levels. However, given the large number of tests performed, this approach is underpowered for identifying SNPs with small effect sizes. In addition, it does not account for genetic interactions, i.e., causal effects that are only observed when specific combinations of mutations and/or non-mutations are jointly present. In order to address these limitations, we also carried out RF analysis to identify combinations of SNPs contributing to variation in salivary testosterone in infancy.

To gain a better understanding of the power of our univariate genetic association analysis, we generated power plots using different SNP MAFs and effect sizes (beta).

We used the same number of subjects (and proportion of MZs, DZs, and singletons) as our real data and similar scale of effect size, mean, variance, and additive genetic and shared environment effects estimated from the real data. Ten thousand simulations were done for each scenario and the significance level was set to 0.05/500, which is similar to our data after Bonferroni correction.

### Random forests

Random forest analysis was implemented using R software. The result of RF analysis reflects joint and conditional effects of multiple variables. As variables with strong effects may mask weaker, yet important effects, we removed the effects of log(10)transferrin, age since DOB, 5 min APGAR score, and the first three principle components (for males) and log(10)transferrin, age since DOB, and the first three principle components (for females). Specifically, we (1) used the residual from the regression Testosterone ~log(10)transferrin + age since DOB + 5 min APGAR score (males only) + PC1 + PC2 + PC3 as the new response, and (2) used the residual from the regression SNPs ~log(10)transferrin + age since DOB + 5 min APGAR score (males only) + PC1 + PC2 + PC3 as the new predictor variables for the RF analysis. The number of randomly preselected variables (*m_try_*) was set to the number of SNPs divided by 3 (*p*/3) as recommended by Liaw and Wiener (38). We also examined the error rate for *m_try_* = 2*p*/3, 0.5*p*/3, and *p*. Error rate was highly similar across different *m_try_*, but *p*/3 produced the lowest error rate. The number of trees (*ntree*) was set to 2000. RF analysis was run 50 times, each run using different seeds for random number generation (RNG). In each run, we recorded the 30 SNPs with the highest variable importance. After all 50 runs were completed, we identified those SNPs, which appeared in the “top 30 list” in every 1 of 50 runs. Because RF analysis is not set-up to treat twins as a repeated measure, we randomly picked one twin from each twin pair to ensure that no related subjects were included in the RF analysis.

## Results

Mean ± SD concentration of salivary testosterone was 40.39 ± 13.39 pg/ml in males and 39.70 ± 16.64 pg/ml in females and did not differ significantly (*p* = 0.73). This is likely a consequence of differing correlations between salivary testosterone and serum testosterone in males and females. According to Salimetrics, the relationship between serum and saliva for males as determined by linear regression is *y* (total serum testosterone in nanogram per milliliter) = 0.2421 + 0.0496 × *x* (salivary testosterone in picogram per milliliter). The linear regression equation for females is *y* (total serum testosterone in nanogram per milliliter) = 0.1415 + 0.0055 × *x* (salivary testosterone in picogram per milliliter). Assuming that the relationship between total serum testosterone and salivary testosterone in infants is similar to that seen in adults; we would estimate a mean serum level of 2.25 ng/ml for male infants and 0.36 ng/ml for female infants, which is comparable to published reports on serum testosterone levels in this age range (39, 40).

Mean ± SD for transferrin was 0.844 ± 0.93 mg/dl in males and 0.91 ± 0.98 mg/dl in females. A moderate correlation between transferrin and testosterone was observed in both males and females (*r* = 0.28, *p* = 0.002 and *r* = 0.43, *p* < 0.001, respectively). Transferrin levels were higher than expected, which raises the possibility of blood contamination. We addressed this issue in two ways. (1) In all our primary analyses, we included transferrin as a covariate. Specifically, we adjusted for log(10)transferrin as the untransformed variable showed high levels of skewness and kurtosis. (2) For the environmental association analysis, univariate genetic analyses, and RF analysis, we also performed sensitivity analyses in infants with transferrin levels <0.50 mg/dl [cut-off based on the recommendation of Granger et al. (41)]. For ICC and heritability estimation, we did not perform sensitivity analyses due to insufficient sample size.

### Environmental association analysis

The final model for males using adaptive Lasso included 5 min APGAR score in addition to the fixed variables, log(10)transferrin, and age since DOB. Bootstrapping supported the importance of 5 min APGAR score in that it was selected in 837 out of 1000 tests (see Table 3). A linear mixed effect model including these variables explained approximately 32% of the variance in salivary testosterone. Examination of a linear mixed effect model including all predictors confirmed that 5 min APGAR score was the only major demographic or medical history variable examined which significantly predicted salivary testosterone in males after Bonferroni correction (0.05/29 predictors = 0.0017) and explained approximately 15% of the variance. In males with transferrin levels <0.50 mg/dl, a linear mixed model including age since DOB and 5 min APGAR score explained 22% of the variance in salivary testosterone (see Table 4). The final model for females using adaptive Lasso only included the fixed variables, log(10)transferrin, and age since DOB. A mixed effect model including these variables explained approximately 18% of the variance in salivary testosterone. Examination of a mixed effect model including all predictors confirmed that none of the demographic or medical history variables examined significantly predicted salivary testosterone in females after Bonferroni correction (0.05/29 predictors = 0.0017). In females with transferrin levels <0.50 mg/dl, a mixed model including age since DOB explained 1% of the variance in salivary testosterone (see Table 5). Given the converging evidence for the importance of 5 min APGAR score on salivary testosterone in males, it was included as a covariate in subsequent analyses.

**Table 3:** **Bootstrapping results**.

	Male	Female
Transferrin^a^ (fixed)	1000	1000
Age since DOB (fixed)	1000	1000
NICU Stay >24 h	43	26
Birth weight	152	84
Gestational age birth	54	28
Maternal ethnicity (White vs. Black)	122	114
Maternal ethnicity (White vs. Asian)	132	3
Maternal ethnicity (White vs. American Indian)	83	
Paternal ethnicity (White vs. Black)	122	9
Paternal ethnicity (White vs. Asian)	8	22
Maternal education	49	23
Paternal education	92	17
Maternal age	110	12
Paternal age	118	19
Maternal psych history	111	6
Paternal psych history	184	11
Income (low vs. middle)	48	141
Income (low vs. high)	410	35
Income (low vs. missing)	39	379
5-Min APGAR	837	20
Gestation number	103	43
C-section	227	32
Smoking	126	5

*^a^Log(10)transferrin*.

**Table 4:** **Association of salivary testosterone with demographic and medical history variables in males**.

Model	*R*^2^	Predictors	Beta	Sig	*r*^2^
Mixed effect model (selected variables)	0.32	Intercept	110.07		
		Transferrin^a^	17.36	<0.001	0.24
		Age since DOB	−5.84	0.08	0.02
		5 Min APGAR	−5.26	<0.001	0.08
Full mixed effect model	0.39	Intercept	183.68		
		Transferrin^a^	17.89	<0.001	0.23
		Age since DOB	−7.99	0.02	0.04
		NICU >24 h	−2.87	0.44	<0.01
		Birth weight	0.00	0.49	0.01
		Gestational age birth	−0.10	0.40	0.02
		Mat ethnicity		0.05	
		White	−14.60		0.20
		Black	−12.56		0.12
		American Indian	−25.34		0.05
		Pat ethnicity		0.51	
		White	2.99		<0.01
		Black	0.13		<0.01
		Mat education	−0.05	0.94	<0.01
		Pat education	−0.64	0.35	0.02
		Mat age	−0.24	0.55	<0.01
		Pat age	0.31	0.50	0.02
		Mat psych history	2.71	0.51	<0.01
		Pat psych history	−6.07	0.17	0.02
		Income		0.08	
		High	3.85		0.02
		Middle	−0.71		<0.01
		Missing	7.78		<0.01
		5-Min APGAR	−7.57	<0.001	0.15
		Gestation number	−3.18	0.38	0.01
		C-section	−3.34	0.18	0.01
		Mat smoking	2.53	0.60	<0.01
Reduced mixed effect model in infants with transferrin <0.5 ng/dl	0.22	Intercept	105.03		
		Age since DOB	−2.93	0.50	<0.01
		5-Min APGAR	−7.07	<0.001	0.22

*^a^Log(10)transferrin*.

**Table 5:** **Association of salivary testosterone with demographic and medical history variables in females**.

Model	*R*^2^	Predictors	Beta	Sig	*r*^2^
Mixed effect model (selected variables)	0.18	Intercept	57.37		
		Transferrin^a^	14.24	<0.001	0.15
		Age since DOB	−4.86	0.28	0.01
Full mixed effect model	0.41	Intercept	70.37		
		Transferrin^a^	15.71	<0.001	0.16
		Age since DOB	−5.73	0.20	0.01
		NICU stay >24 h	−6.41	0.27	0.02
		Birth weight	−0.01	0.08	0.07
		Gestational age birth	0.06	0.72	<0.01
		Mat Ethnicity		0.24	
		White	−5.03		0.01
		Black	3.01		<0.01
		Pat ethnicity		0.16	
		White	14.02		0.11
		Black	8.37		0.03
		Mat education	0.43	0.58	<0.01
		Pat education	0.24	0.73	<0.01
		Mat age	0.16	0.74	<0.01
		Pat age	−0.21	0.62	<0.01
		Mat psych history	0.18	0.96	<0.01
		Pat psych history	−2.67	0.64	<0.01
		Income		0.004	
		High	−2.59		<0.01
		Middle	2.87		<0.01
		Missing	−28.57		0.05
		5-Min APGAR	−0.97	0.66	<0.01
		Gestation number	−6.03	0.23	0.03
		C-section	−0.26	0.94	<0.01
		Mat smoking	1.53	0.79	<0.01
Reduced mixed effect model in infants with transferrin <0.5 ng/dl	0.01	Intercept	17.89		
		Age since DOB	3.48	0.22	0.01

*^a^Log(10)transferrin*.

### Intraclass correlation coefficient and heritability estimation

**Figure 3:**
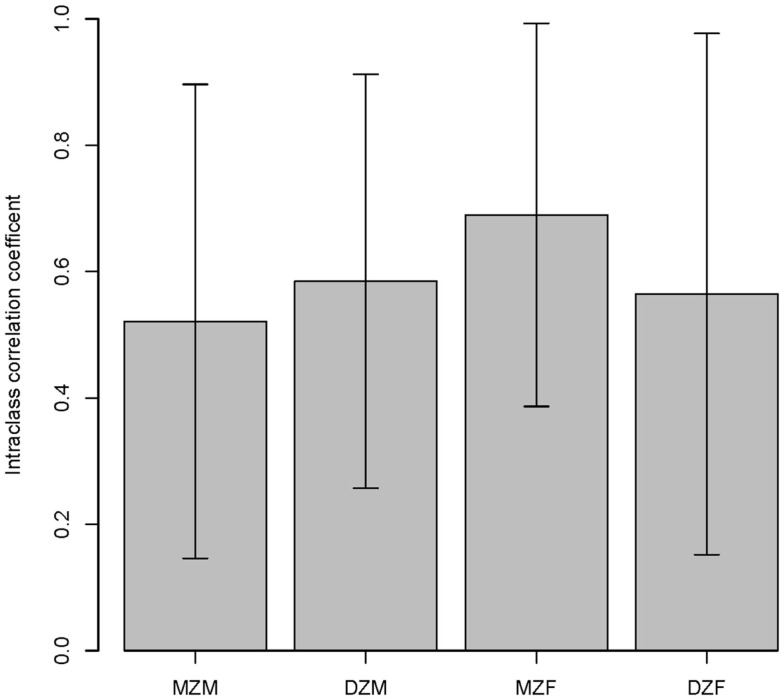
**Intraclass correlations within twin pairs are shown**. MZM (monozygotic male), DZM (dizygotic male), MZF (monozygotic female), and DZF (dizygotic female).

The intraclass correlations for MZ and DZ twins are shown in Figure 3 and suggest a high environmental component and a low genetic component for both sexes. The ACE model confirmed that the majority of variation in salivary testosterone was explained by shared environmental factors in both sexes (see Table 6).

**Table 6:** **Univariate genetic model**.

	Variance explained (%)	*p*-value
Males
A	0	0.81
C	62.7	<0.001
Females
A	19.1	0.36
C	41.8	0.19

*Percentages of phenotypic variation explained by additive genetic (A) and shared environmental (C) factors*.

### Univariate genetic association analysis

**Figure 4:**
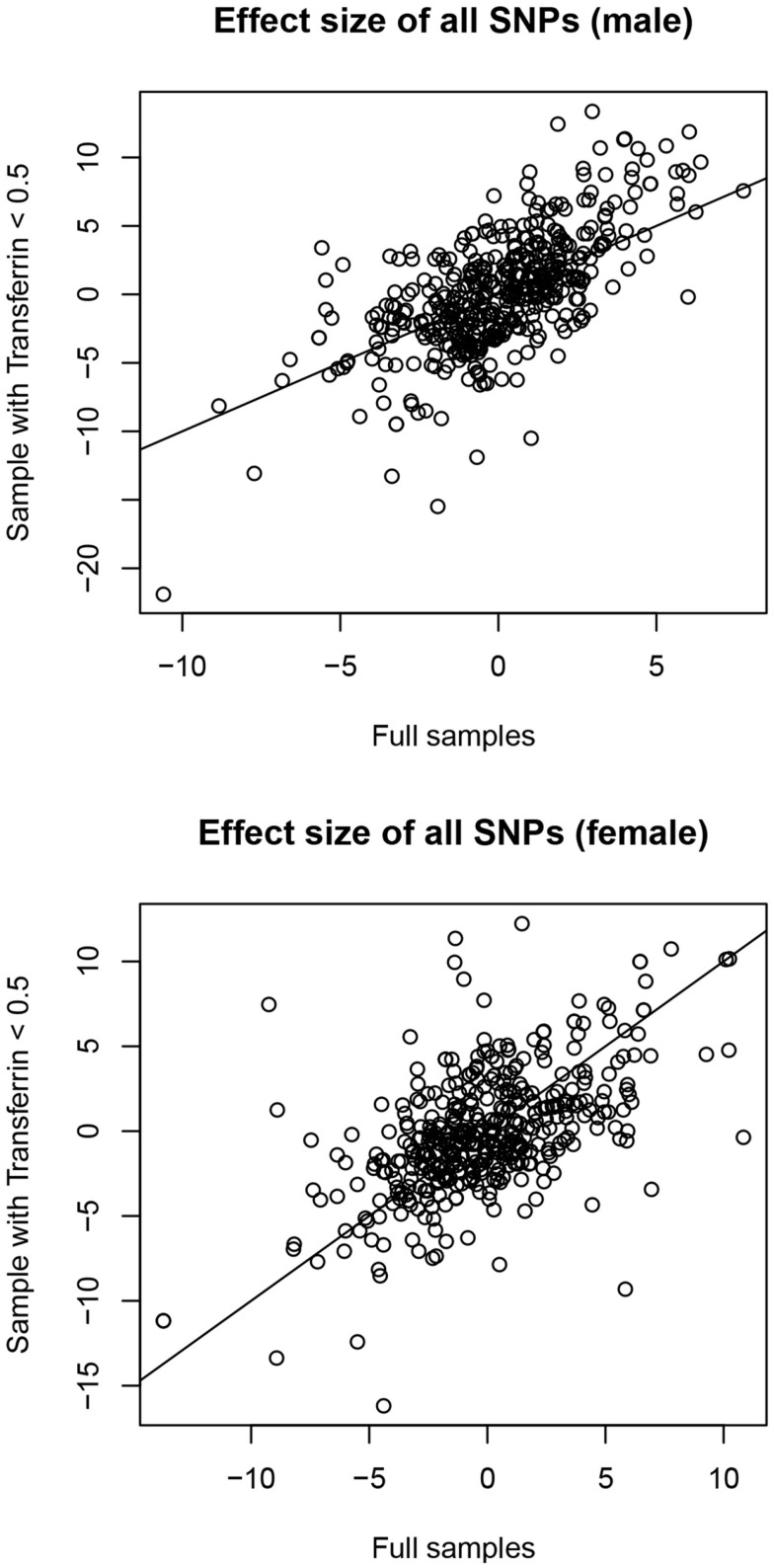
**Correlation between effect sizes in full samples [correcting for log(10)transferrin] and subsamples with transferrin levels <0.50 mg/dl**.

**Figure 5:**
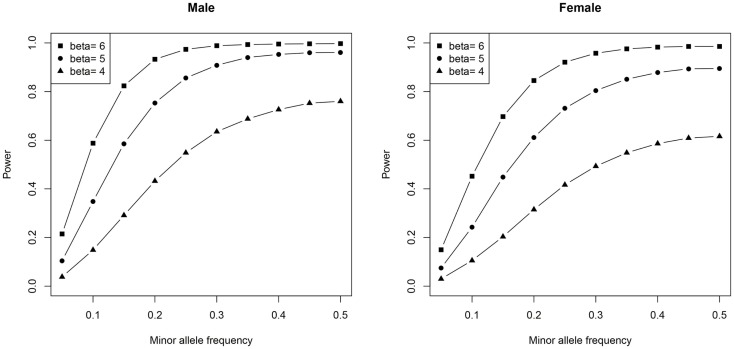
**Plots demonstrating the relationship between statistical power, minor-allele frequencies, and effect size are shown**.

In males, no SNPs were significantly associated with salivary testosterone levels after adjusting for FDR in the full sample. The most significant association was for rs10923844, a variant of unknown function located downstream of *HSD3B1* and *HSD3B2* (adjusted *p*-value = 0.07, unadjusted *p* = 0.0002). This same SNP had an unadjusted *p*-value of 0.02 in the subsample of males with transferrin <0.50 mg/dl. Effect sizes for this variant were highly similar in the total male sample [adjusting for log(10)transferrin] and the subsample of males with transferrin <0.50 mg/dl (Beta = 6.26 and 6.02, respectively). Indeed, effect sizes for the full male sample are highly similar to the corresponding effect sizes in the subset of males with transferrin <0.50 mg/dl (see Figure 4). None of the previous GWAS hits for serum testosterone levels in adult males were significantly associated with salivary testosterone in infant males before or after adjusting for FDR in either the full sample or the subset of males with transferrin <0.50 mg/dl. In females, no SNPs were significantly associated with salivary testosterone levels after adjusting for FDR. The most significant associations were for two SNPs located upstream of *ESR1* (rs3407085 and rs2295190) within the intron region of *SYNE1* (*spectrin repeat containing, nuclear envelope (1)*; adjusted and unadjusted p-values were 0.09 and 0.0004 for both variants (these variants are in complete linkage disequilibrium within our sample). Both variants had an unadjusted p-value of 0.04 in the subsample of females with transferrin <0.50 mg/dl. Effect sizes were highly similar for the total female sample [adjusting for log(10)transferrin] and the subsample of females with transferrin <0.50 mg/dl (Beta = −13.7 and −11.2, respectively), though we note that effect sizes for the full female sample do not correspond as well to the effect sizes in the reduced sample as compared to males (See Figure 4). Full results are available as supplemental data. Power calculations indicate that theoretically, our analysis is well-powered to identify variants with Beta values >6 in the MAF range examined (see Figure 5).

### Random forests

Tables 7 and 8 display the top SNPs in males and females, respectively, defined as the intersection of the top 30 variables obtained from each of 50 RF runs. Also included in the table are the beta.hat, likelihood ratio, and unadjusted and FDR adjusted *p*-values from the univariate association tests. In males, regulators of reproductive function appear to play an important role. Specifically, multiple variants in or near *LHCGR* were consistently selected as high importance. Multiple SNPs in genes related to cholesterol production, transport, and removal were also selected. These genes included *DHCR7*, *TSPO*, *CYP7A1*, and *POR*. The top hit from the univariate analyses (rs10923844) was selected, as were two variants near the Xp22 loci identified in GWAS studies of serum testosterone in adult males. Analyses in the subset of males with transferrin <0.50 mg/dl supported the importance of regulators of reproductive function and cholesterol-related genes. Five SNPs were selected as high importance in both the full and reduced male sample; these were rs4952922, rs10495960, and rs2301267 (all in/near *LHCGR*), rs47340 (*TSPO*), and rs74091680 (*HSD17B6*). In females, genes related to estrogen signaling appear to play an important role. Specifically, variants in *ESR1*, *CYP19A1*, *CYP1B1*, and *HSD17B8* were consistently selected as high importance in the full sample. Analyses in the subset of females with transferrin <0.50 mg/dl supported the importance of estrogen signaling, although the specific SNPs differed. Two SNPs were selected as high importance in both the full and reduced female sample; these were rs4149448 (*SULT2A1*) and rs4659175 (*HSD3B2*).

**Table 7:** **Random forest results males**.

SNP	beta.hat	lr	mChr	mPos	pval	pval.bh	Index gene	Function class
**FULL SAMPLE**								
rs10923844	6.26	13.42	chr1	120000000	0.0002	0.07	*HSD3B1*	Downstream
rs2301267	5.66	12.38	chr2	48984391	0.0004	0.07	*LHCGR*	Upstream (*LHCGR*) intronic (*STON1-GTF2A1L*)
rs11897846	2.94	3.29	chr2	48956512	0.07	0.66	*LHCGR*	Intronic
rs10495960	3.97	3.26	chr2	48960032	0.07	0.66	*LHCGR*	Intronic (*LHCGR*) missense (*GTF2A1L*)
rs4952922	4	3.29	chr2	48961396	0.07	0.66	*LHCGR*	Intronic
rs988328	−6.6	8.42	chr6	152000000	0.004	0.35	*ESR1*	Intronic
rs4728533	1.82	0.75	chr7	75586536	0.38	0.90	*POR*	Intronic
rs10504255	3.63	3.64	chr8	59398461	0.06	0.66	*CYP7A1*	Downstream
rs1004467	4.04	2.85	chr10	105000000	0.09	0.66	*CYP17A1*	Intronic
rs12419334	4.62	7.34	chr11	71139472	0.007	0.38	*DHCR7*	Downstream
rs12797951	4.17	6.26	chr11	71143266	0.01	0.57	*DHCR7*	Downstream
rs74091680	7.75	7.47	chr12	57154822	0.006	0.38	*HSD17B6*	Intronic
rs47340	5.66	12.8	chr22	43562829	0.0003	0.07	*TSPO*	Downstream (*TSPO*) 3 prime UTR (*TTLL12*)
rs139036121	−0.21	0.03	chrX	8912628	0.87	0.96	*Xp22 near GWAS hit*	Intergenic b/t *FAM9A* and *FAM9B*
rs5934508	−0.1	0.01	chrX	8918776	0.92	0.97	*Xp22 near GWAS hit*	Intergenic b/t *FAM9A* and *FAM9B*
**SUBSET WITH TRANSFERRIN <0.50 ng/dl**								
rs56058466	9.15	6.42	chr2	38328870	0.01	0.21	*CYP1B1*	Upstream
rs4952922	11.36	17.97	chr2	48961396	0.00002	0.006	*LHCGR*	Intronic
rs10495960	11.30	17.81	chr2	48960032	0.00002	0.0006	*LHCGR*	Intronic (*LHCGR*) missense (*GTF2A1L*)
rs2301267	7.37	13.36	chr2	48984391	0.0003	0.03	*LHCGR*	Upstream
rs4245818	8.94	10.78	chr2	48985607	0.001	0.06	*LHCGR*	Upstream (*LHCGR)* Intronic (*STON1-GTF2A1L*)
rs11682325	9.86	6.56	chr2	48899807	0.01	0.21	*LHCGR*	Downstream (*LHCGR*
								Intronic (*STON1-GTF2A1L*)
rs2031367	8.74	7.83	chr6	87807180	0.01	0.17	*CGA*	Upstream
rs6937568	12.46	5.22	chr6	152153964	0.02	0.28	*ESR1*	Intronic
rs62442039	13.38	12.90	chr6	152158090	0.0003	0.03	*ESR1*	Intronic
rs10954724	−4.19	3.16	chr7	75597545	0.08	0.46	*POR*	Intronic
rs17148944	10.69	5.96	chr7	75601867	0.01	0.23	*POR*	Intronic
rs800667	2.91	0.41	chr7	99447241	0.52	0.81	*CYP3A43*	Synonymous
rs881671	−21.87	12.79	chr8	59417107	0.0003	0.03	*CYP7A1*	Upstream
rs8190495	5.80	7.09	chr9	99061884	0.01	0.18	*HSD17B3*	Intronic
rs8190478	6.23	9.07	chr9	99064883	0.003	0.12	*HSD17B3*	Upstream
rs74091680	7.59	2.03	chr12	57154822	0.15	0.55	*HSD17B6*	Intronic
rs2277339	2.67	0.47	chr12	57146069	0.49	0.82	*HSD17B6*	Upstream (*HSD17B6*) missense (*PRIM1*)
rs47340	6.61	6.83	chr22	43562829	0.01	0.20	*TSPO*	Downstream (*TSPO*) 3 prime UTR (*TTLL12*)

**Table 8:** **Random forest results females**.

SNP	beta.hat	lr	mChr	mPos	pval	pval.bh	Index gene	Function class
**FULL SAMPLE**								
rs4659175	−3.44	2.08	chr1	119956473	0.15	0.80	*HSD3B2*	Upstream
rs4952222	1.58	0.13	chr2	31799863	0.72	0.97	*SRD5A2*	Intronic
rs56058466	6.90	4.73	chr2	38328870	0.03	0.52	*CYP1B*	Upstream
rs1547387	−8.21	5.75	chr6	33169895	0.02	0.39	*HSD17B8*	Upstream (*HSD17B8*) synonymous (*SLC39A7)*
rs11155820	1.43	0.29	chr6	152204210	0.59	0.96	*ESR1*	Intronic
rs62443560	−13.68	6.59	chr6	152190476	0.01	0.35	*ESR1*	Intronic
rs2982683	2.95	1.37	chr6	152298435	0.24	0.91	*ESR1*	Intronic
rs17081685	1.46	0.14	chr6	152116655	0.71	0.97	*ESR1*	Intronic
rs3798758	−5.50	1.37	chr6	152421854	0.24	0.91	*ESR1*	3 prime UTR
rs2899472	−0.46	0.04	chr15	51516055	0.85	0.99	*CYP19A1*	Intronic
rs9939740	−2.96	1.54	chr16	82121981	0.21	0.88	*HSD17B2*	Intronic
rs6259	−4.61	1.56	chr17	7536527	0.21	0.88	*SHBG*	Missense
rs4149448	5.52	2.68	chr19	48386357	0.10	0.69	*SULT2A1*	Intronic
rs138929	−4.78	3.44	chr22	43562439	0.06	0.58	*TSPO*	Downstream
rs57484470	5.01	0.87	chr22	43545077	0.35	0.92	*TSPO*	Upstream
rs6971	−4.70	2.89	chr22	43558926	0.09	0.68	*TSPO*	Missense
rs7058445	−2.03	0.67	chrX	7172508	0.41	0.94	*STS*	Intronic
**SUBSET WITH TRANSFERRIN < 0.50 ng/dl**								
rs4659175	−3.76	2.32	chr1	119956473	0.13	1	*HSD3B2*	Upstream
rs10923844	−4.58	2.84	chr1	120059500	0.09	0.90	*HSD3B1*	Downstream
rs232535	−0.60	0.04	chr2	38332303	0.85	1	*CYP1B1*	Upstream
rs162557	−2.85	1.17	chr2	38305451	0.28	1	*CYP1B1*	Upstream
rs28585480	−7.35	7.02	chr4	74290918	0.01	0.86	*ALB*	Downstream
rs2747653	6.47	−1.19	chr6	152446057	1.00	1	*ESR1*	Downstream (*ESR1*) intronic (*SYNE1*)
rs10224569	10.19	0.33	chr7	99248304	0.57	1	*CYP3A5*	Intronic
rs12537277	−0.61	0.04	chr7	75588704	0.84	1	*POR*	Intronic
rs10135310	9.95	−0.28	chr14	64574140	1.00	1	*ESR2*	Downstream (*ESR2*) intronic (*SYNE2*)
rs2781377	10.02	−1.42	chr14	64560092	1.00	1	*ESR2*	Downstream (*ESR2*) stop gained (*SYNE2*)
rs41334947	10.02	−1.42	chr14	64560091	1.00	1	*ESR2*	Downstream (*ESR2*) missense(*SYNE2*)
rs57018718	10.02	−1.42	chr14	64594019	1.00	1	*ESR2*	Downstream (*ESR2*) intronic (*SYNE2*)
rs2414095	−6.29	0.57	chr15	51524292	0.45	1	*CYP19A1*	Intronic
rs12591359	2.23	0.68	chr15	51539368	0.41	1	*CYP19A1*	Intronic
rs4149448	4.08	2.03	chr19	48386357	0.15	1	*SULT2A1*	Intronic
rs2910400	3.56	1.41	chr19	48394042	0.24	1	*SULT2A1*	Upstream

## Discussion

This study provides the first detailed analysis of environmental and genetic contributors to variation in salivary testosterone during the minipuberty, a developmental period that plays a critical role in genital development and sexual differentiation of the brain. As such, it should be considered exploratory and requires replication. Twin modeling indicated that individual variability in testosterone levels in the minipuberty is primarily explained by environmental factors. In terms of specific environmental contributors, we observed a consistent and robust association between 5 min APGAR scores and salivary testosterone levels in males. In terms of specific genetic contributors, univariate tests did not reveal any variants significantly associated with salivary testosterone after adjusting for FDR. However, we note that this approach is underpowered for identifying SNPs with small effect sizes. The top hit in males was rs10923844, an SNP of unknown function located downstream of *HSD3B1* and *HSD3B2*. The top hits in females were two SNPs located upstream of *ESR1* (rs3407085 and rs2295190). RF analysis, which reflects joint and conditional effects of multiple variables, including those with small individual effect sizes, suggests that genes involved in regulation of reproductive function and cholesterol production, transport, and removal are involved in individual variation in salivary testosterone in males, while genes involved in estrogen signaling are important in females.

### Major demographic and medical history variables and salivary testosterone

Higher APGAR scores taken 5 min post-birth were associated with lower salivary testosterone levels in males during the minipuberty. APGAR scores were originally designed to quickly evaluate a newborn’s physical condition and to identify any immediate need for extra medical or emergency care. The APGAR score includes five components: heart rate, respiratory effort, muscle tone, reflex irritability, and color, each of which is given a score of 0, 1, or 2 with 2 being the best score (42). A number of factors may influence an APGAR score, including hypoxia, exposure to drugs, trauma, congenital anomalies, infections, and hypovolemia (43). While primarily conceived as a measure of short peripartum stress, some researchers have suggested that it also indexes a suboptimal fetal environment (44). Studies in rodent models provide compelling evidence that the stress response induced by physical or emotional challenges in fetal life affects later reproductive function (45–47). Regarding the HPG axis, maternal stress during pregnancy disrupts the prenatal surge of testosterone that normally occurs in the developing male rat (48, 49) and is associated with reduced testosterone levels in adult rats (50). No studies have examined stress response during labor and delivery and its impact on reproductive function. Physical stressors associated with lower APGAR scores may explain the association between 5 min APGAR scores and salivary testosterone in males observed in our study, although the direction of effect is opposite to that reported for prenatal stress in rodents. We note that while 5-min Apgar score is a valid predictor of neonatal mortality, its predictive value for other outcomes continues to be debated (43). Never-the-less, this scoring system remains the only widely used and accepted tool for assessing the vitality of newborn infants across the world. We also note that the majority of children in our sample had 5-min Apgar scores between 7 and 10, which is considered normal, with only a few falling in the intermediate range (scores between 4 and 6). Additional research is necessary to replicate these findings, determine if they extend to individuals with lower APGAR scores, and understand underlying mechanisms. None of the major demographic or maternal history variables examined in this study impacted salivary testosterone levels in females.

### Heritability of salivary testosterone

Our results suggest that individual variability in salivary testosterone levels in the minipuberty is predominantly explained by environmental factors. Our findings are similar to those reported by Caramaschi et al. (28) in older infants, but contrast with studies in adolescents and adults, which show high heritability, especially in males (17–23). Caramaschi et al. (28) suggested that the pubertal rise in male testosterone levels has a strong genetic component and that testosterone levels are less influenced by genetic factors when male and female levels are similar and very low as is the case in later infancy. Salivary testosterone levels in our study were significantly higher than those reported by Caramaschi et al. (28): 40.39 vs. 9.60 pg/ml in boys and 39.70 vs. 8.93 pg/ml in girls, but are lower than those reported for adults: 163.81 and 60.86 pg/ml for males and females, respectively (51). Salivary testosterone did not differ significantly between males and females in our sample, but estimated serum levels exhibited a sex difference, which is comparable to published reports on serum testosterone levels in this age range (39, 40). Therefore, our results suggest that environmental factors play a stronger role than genetic factors during the minipuberty despite moderately high testosterone levels and sex differences in serum testosterone. Identifying the environmental factors involved is an important area for future research.

### Genetic factors influencing salivary testosterone

Although our heritability analysis indicated that environmental risk factors play a larger role in determining individual variation in salivary testosterone during the minipuberty than genetic factors, deciphering the genetic component is still important in terms of understanding underlying biochemical pathways. We were also mindful of the fact that heritability by itself does not provide information about the genetic architecture of traits. In principle, a trait with a low heritability can have a single locus that causes variation and a trait with a high heritability can have hundreds of contributing loci (52). The presence of gene–environment correlations and interactions also introduces systematic biases in heritability estimates made under the independence assumption in twin studies, which can result in an underestimation of the genetic component (53) For all these reasons, we performed univariate genetic association analyses and RF analyses of SNPs within ±5 kb of genes involved in testosterone synthesis, transport, signaling, and metabolism for their relationship with salivary testosterone.

No SNPs were significantly associated with salivary testosterone levels after adjusting for FDR in the full male sample or the full female sample. Simulations indicated that theoretically we were well-powered to detect significant SNPs with Beta values >6 within the range of minor-allele frequencies we examined. Thus, our study suggests that common variants with large effect sizes do not play a role in individual differences in salivary testosterone in infants, at least within the genes examined. The top hit in males was rs10923844, a variant of unknown function located downstream of *HSD3B1* and *HSD3B2*, two genes that play a critical role in steroidogenesis. This variant was also identified as an important variable in the RF analysis. Both isoforms of 3-beta-HSD convert pregnenolone to progesterone, 17α-hydroxypregnenolone to 17α-hydroxyprogesterone, dehydroepiandrosterone (DHEA) to androstenedione, and androstenediol to testosterone. Type 1 is primarily expressed in the placenta and non-steroidogenic tissues, while type 2 is primarily expressed in the adrenals and gonads (54). The top hits in females were two SNPs located upstream of *ESR1* (rs3407085 and rs2295190), the gene coding for estrogen receptor alpha, one of two main types of estrogen receptor.

It is increasingly recognized that individual variation in complex phenotypes results, in part, from the joint and conditional effects of many common SNPs whose individual effect sizes are relatively small, making them difficult to identify via standard univariate analyses (55, 56). RF analysis represents a promising approach to this problem and is well-suited to high-dimension, low-sample-size data typical of genetic association studies. RF analysis suggested that regulators of reproductive function and genes related to cholesterol production, transport, and removal play an important role in salivary testosterone levels in males. Five SNPs were selected as high importance in both the full and reduced male sample and thus are particularly promising candidates for follow-up. rs4952922, rs10495960, and rs2301267 are all located in/near *LHCGR*, which codes for the LH/choriogonadotropin receptor. rs47340 is located downstream of *TSPO*, which codes for a protein that interacts with StAR (steroidogenic acute regulatory protein) to transport cholesterol into mitochondria to permit steroid synthesis [data on gene functions from www.genecards.org (57), SNP locations from dbSNP (58) and UCSC genome browser (59)]. rs74091680 is an intronic variant in *HSD17B6*, which codes for an enzyme involved in androgen catabolism. Specifically, the oxidoreductase activity can convert 3 alpha-adiol to dihydrotestosterone, while the epimerase activity can convert androsterone to epi-androsterone. Interestingly, two variants near the Xp22 loci identified in GWAS studies of serum testosterone in adult males (24, 25) were selected in the primary RF analysis. This suggests that some genetic factors influencing testosterone levels are active throughout the lifespan, although this finding was not recapitulated in the subsample analysis.

In females, RF analysis suggested that genes related to estrogen signaling play an important role. Specifically, variants in/near *ESR1*, *CYP19A1*, *CYP1B1*, and *HSD17B8* were consistently selected as high importance in the full sample. *ESR1* codes for estrogen receptor alpha, one of two main types of estrogen receptor. *CYP19A1* codes for aromatase, the enzyme responsible for the conversion of androgens into estrogens. *CYP1B1* codes for an enzyme that metabolizes multiple compounds, including 17β-estradiol. *HSD17B8* inactivates estrogens and androgens, with high activity toward estrogen and low activity toward testosterone. It can also synthesize estradiol from estrone. Analyses in the subset of females with transferrin <0.50 mg/dl supported the importance of estrogen signaling, although the specific SNPs implicated differed. Two SNPs were selected as high importance in both the full and reduced female sample; these were rs4149448 (*SULT2A1*) and rs4659175 (*HSD3B2*). *SULT2A1* codes for a protein, which catalyzes the sulfation of steroids and bile acids in the liver and adrenal glands, and may have a role in the inherited adrenal androgen excess in women with polycystic ovary syndrome (60). *HSD3B2* codes for an enzyme with a critical role in the biosynthesis of all classes of steroid hormones.

### Limitations

The primary limitation of the current study is the use of salivary testosterone levels rather than plasma testosterone levels. While plasma and serum are the traditional matrices for the determination of endocrine parameters including testosterone, saliva offers a non-invasive and stress-free alternative, which enjoys widespread acceptance and has been used for over 40 years (61). In men, salivary testosterone is strongly correlated with the free fraction of testosterone in serum (*r* = 0.64–0.97) (51, 62–64). In women, reported correlations are more moderate (*r* = 0.37–0.85) (51, 63, 65). There is no *a priori* reason to presume that correlations between serum and saliva are different in the neonate, although this has not been directly tested. Collection of saliva maximized parental acceptability and minimized child distress. It is unlikely that we would have achieved a reasonable sample size if we opted to use blood. Substitution of saliva assay results for serum values is known to underestimate testosterone–behavior associations, primarily in females, and this problem may also be applicable to the environmental and genetic associations, which were the focus of this study. Null results, especially in females, should be treated with caution.

We also observed higher levels of transferrin in saliva than expected and moderate correlations between transferrin and testosterone in both sexes (stronger in females), which raises the possibility of blood contamination. For comparison, Granger et al. (41), in a study of children between 6 and 13 years of age, reported a mean ± SD for transferrin of 0.37 ± 0.46 mg/dl and a correlation with testosterone of 0.058. Unfortunately, although the transferrin assay (66) has been available for over a decade, it has not been routinely used in studies of infants making it difficult to determine if the levels that we observed are typical for this age or not. Reassuringly, the mean ± SD for salivary testosterone in our study was highly similar to that reported by other studies in our age range (11) and estimated serum levels are comparable to published reports on serum testosterone in this age range (39, 40). We also addressed this issue statistically. In all our primary analyses, we included transferrin as a covariate. In addition, for the environmental association analysis, univariate genetic analyses, and RF analysis, we performed sensitivity analyses in infants with transferrin levels <0.50 mg/dl (cut-off based on the recommendation of Granger et al. (41). In general, relationships identified in the full sample were also present in the subsample with transferrin levels <0.50 mg/dl. Finally, we note that collecting samples on multiple days would have allowed a more precise evaluation of individual differences in testosterone levels, but was deemed impractical. The sample size for twin modeling was also somewhat underpowered.

## Conclusion

Despite these limitations, the current study provides novel information about the environmental and genetic contributors to testosterone levels in the early post-natal period. In contrast to the strong genetic contributions observed during puberty, transient activation of the HPG axis in the early post-natal period appears to be heavily influenced by environmental factors. We identified 5 min APGAR score as a significant predictor of salivary testosterone levels in males. Further research is needed to elucidate the biological mechanisms underlying this relationship. Our study also suggests that genetic variants in regulators of reproductive function and cholesterol play an important role in salivary testosterone levels in males, while genes related to estrogen signaling play an important role in females. All results require replication, preferably with serially collected serum samples. Ideally, such a study would also include measures of prenatal testosterone in amniotic fluid or through cordocentesis, providing insight into the genetic architecture of the prenatal testosterone surge as well as the neonatal surge.

## Author Contributions

Kai Xia, Yang Yu, Mihye Ahn, Hongtu Zhu, and Fei Zou made substantial contributions to the analysis and interpretation of data for this project. John H. Gilmore made substantial contributions to the acquisition of data for this project. Rebecca C. Knickmeyer made substantial contributions to the conception and design of the work; as well as the acquisition, analysis, and interpretation of data. Kai Xia, Yang Yu, Mihye Ahn, and Rebecca C. Knickmeyer drafted the manuscript. All coauthors revised the manuscript for important intellectual content, and approved the final version to be published. RK agreed to be accountable for all aspects of the work in ensuring that questions related to the accuracy or integrity of any part of the work are appropriately investigated and resolved.

## Conflict of Interest Statement

The authors declare that the research was conducted in the absence of any commercial or financial relationships that could be construed as a potential conflict of interest.

## Supplementary Material

The Supplementary Material for this article can be found online at http://www.frontiersin.org/Journal/10.3389/fendo.2014.00187/abstract
