# Supplementary material for: Environmental and Genetic Contributors to Salivary Testosterone Levels in Infants
Source: Front Endocrinol (Lausanne). 2014 Oct 30;5:187. doi: 10.3389/fendo.2014.00187 (PMC4214198; doi:10.3389/fendo.2014.00187)
Supplement: Supplementary file 1 [file Presentation_1.ZIP › Demographic Interview.PDF]

**Mother Initials:**				**Visit Date (mm dd, yyyy):**			

**Subject #:**					**—**		**Baby #:**		**Visit #:**	**1**

DEMOGRAPHICS (Mother and Father)

Maternal Age: ______ Maternal Date of Birth: __ __/__ __/__ __ __ __

Maternal Ethnicity:

White	
Black or African American	
American Indian or Alaskan Native	
Asian	
Native Hawaiian or Other Pacific Islander	

Is Mother Spanish/Hispanic/Latino? Yes____ No____

Paternal Age: ______ Paternal Date of Birth: __ __/__ __/__ __ __ __

Paternal Ethnicity:

White	
Black or African American	
American Indian or Alaskan Native	
Asian	
Native Hawaiian or Other Pacific Islander	

Is Father Spanish/Hispanic/Latino? Yes____ No____

Marital Status

1. Married	
2. Living together	
3. Divorced	
4. Separated	
5. Never married	
6. Widowed	

Comments: ____ (Not for Data Entry)

**Mother Initials:**				**Visit Date (mm dd, yyyy):**			

**Subject #:**					**—**		**Baby #:**		**Visit #:**	**1**

SOCIOECONOMIC INFORMATION (Mother and Father)

Information About the Mother

Education (Total in years & grade/degree):_________

Education Code (Hollingshead):____________

Employment Status: Full Part time Unemployed Disability Student Housewife

Occupation _________________________________ Place of Employment ___________________________________

Years Employed ___________ Income ______________________________

If not employed outside the home, list last paid occupation and dates of employment.

Occupation _______________________________ Dates of Employment ___________________________________

Highest Occupation (Hollingshead Code):___________________

Information About the Father

Education (Total in years & grade/degree):_________

Education Code (Hollingshead):____________

Employment Status: Full Part time Unemployed Disability Student House husband

Occupation _________________________________ Place of Employment ___________________________________

Years Employed _____________ Income ___________________________

If not employed outside the home, list last paid occupation and dates of employment.

Occupation _______________________________ Dates of Employment ____________________________________

Highest Occupation (Hollingshead Code):____________

Other Sources of Income (Specify):__________________

Total Household Income (TINN):___________________
