# Supplementary material for: Environmental and Genetic Contributors to Salivary Testosterone Levels in Infants
Source: Front Endocrinol (Lausanne). 2014 Oct 30;5:187. doi: 10.3389/fendo.2014.00187 (PMC4214198; doi:10.3389/fendo.2014.00187)
Supplement: Supplementary file 1 [file Presentation_1.ZIP › Maternal Medication History.PDF]

**Mother Initials:**				**Visit Date (mm, dd, yyyy):**					
**Baby Initials:**									
**Subject #:**					—		**Baby #:**		**Visit** 1

MEDICATION & DRUG ASSESSMENT - SINCE BECOMING PREGNANT

1. **Antipsychotics** 0=No/Unknown 1=Yes Trimester
   Specify: ___________________________ begin? __1st __2nd __3rd
   stop? __1st __2nd __3rd
2. **Antidepressants** 0=No/Unknown 1=Yes
   Specify: ___________________________ begin? __1st __2nd __3rd
   stop? __1st __2nd __3rd
3. **Benzodiazepines** 0=No/Unknown 1=Yes
   Specify: ___________________________ begin? __1st __2nd __3rd
   stop? __1st __2nd __3rd
4. **Antiparkinsonian** 0=No/Unknown 1=Yes
   Specify: ___________________________ begin? __1st __2nd __3rd
   stop? __1st __2nd __3rd
5. **Antiseizure Medications** 0=No/Unknown 1=Yes
   Specify: ___________________________ begin? __1st __2nd __3rd
   Stop? __1st __2nd __3rd
6. **Steroidal AI Drugs** 0=No/Unknown 1=Yes
   Specify: ___________________________ begin? __1st __2nd __3rd
   stop? __1st __2nd __3rd
7. **Cold Remedy/Antihist/Decong** 0=No/Unknown 1=Yes
   Specify: ___________________________ begin? __1st __2nd __3rd
   stop? __1st __2nd __3rd
8. **Herbals** 0=No/Unknown 1=Yes
   Specify: ___________________________ begin? __1st __2nd __3rd
   stop? __1st __2nd __3rd
9. **Alcohol Drinks Per Week** 0=None/Unknown 1=Yes
   Specify Number (drinks/week) __ __ begin? __1st __2nd __3rd
   stop? __1st __2nd __3rd
10. **Drug Use** 0=No/Unknown 1=Yes
    Specify: ___________________________ begin? __1st __2nd __3rd
    stop? __1st __2nd __3rd
11. **Smoking (cig/day)** 0=None/Unknown 1=Yes
    Specify Number Cigarettes/Day __ __ begin? __1st __2nd __3rd
    stop? __1st __2nd __3rd
12. **Other Medications** 0=No/Unknown 1=Yes
    Specify: ___________________________ begin? __1st __2nd __3rd
    stop? __1st __2nd __3rd
    Specify: ___________________________ begin? __1st __2nd __3rd
    stop? __1st __2nd __3rd
    Specify: ___________________________ begin? __1st __2nd __3rd
