# Supplementary material for: Environmental and Genetic Contributors to Salivary Testosterone Levels in Infants
Source: Front Endocrinol (Lausanne). 2014 Oct 30;5:187. doi: 10.3389/fendo.2014.00187 (PMC4214198; doi:10.3389/fendo.2014.00187)
Supplement: Supplementary file 1 [file Presentation_1.ZIP › Maternal Psych History V1.PDF]

**Mother Initials:**				**Visit Date (mm dd, yyyy):**			

**Subject #:**					—		**Baby #:**		**Visit #:**	1

SCID PSYCHIATRIC ASSESSMENT - MOTHER

Rater Initials: ___________

**Diagnosis**	**History**			
Schizophrenia	1.	Lifetime History	Yes	
			No	
	2.	Present During Past Month	Yes	
			No	
Schizophreniform Disorder	3.	Lifetime History	Yes	
			No	
	4.	Present During Past Month	Yes	
			No	
Schizoaffective Disorder	5.	Lifetime History	Yes	
			No	
	6.	Present During Past Month	Yes	
			No	
Major Depression	7.	Lifetime History	Yes	
			No	
	8.	Present During Past Month	Yes	
			No	
Bipolar Disorder	9.	Lifetime History	Yes	
			No	
	10.	Present During Past Month	Yes	
			No	
Alcohol Dependence	11.	Lifetime History	Yes	
			No	
	12.	Present During Past Month	Yes	
			No	
Alcohol Abuse	13.	Lifetime History	Yes	
			No	
	14.	Present During Past Month	Yes	
			No	

**Mother Initials:**				**Visit Date (mm dd, yyyy):**			

**Subject #:**					**—**		**Baby #:**		**Visit #:**	**1**

SCID/PSYCHIATRIC HISTORY (continued)

**Diagnosis**	**History**		
Drug Dependence	15. Lifetime History	Yes	
		No	
	If Yes, check all that apply:		
	Marijuana		
	Cocaine		
	Opiates		
	PCP		
	Amphetamines		
	Other, Specify: ____________________		
	16. Present During Past Month	Yes	
		No	
	If Yes, check all that apply:		
	Marijuana		
	Cocaine		
	Opiates		
	PCP		
	Amphetamines		
	Other, Specify: ____________________		

**Mother Initials:**				**Visit Date (mm dd, yyyy):**			

**Subject #:**					—		**Baby #:**		**Visit #:**	1

SCID/PSYCHIATRIC HISTORY (continued)

**Diagnosis**	**History**			
Drug Abuse	17.	Lifetime History	Yes	
			No	
	If Yes, check all that apply:			
		Marijuana		
		Cocaine		
		Opiates		
		PCP		
		Amphetamines		
	Other, Specify: ____			
	18.	Present During Past Month	Yes	
			No	
	If Yes, check all that apply:			
		Marijuana		
		Cocaine		
		Opiates		
		PCP		
		Amphetamines		
	Other, Specify: ____			

**Treatment History**		
19. Have you ever been treated by a health care provider for any behavioral or emotional problem?	Yes	
	No	
	If yes: How old were you when you were first treated? (years)	
20. Have you ever been prescribed any antipsychotic medication?	Yes	
	No	
	If yes: Estimate date first prescribed	
	Estimate number of years that you have taken antipsychotic medication.	

**Mother Initials:**				**Visit Date (mm dd, yyyy):**					
**Subject #:**					—		**Baby #:**		**Visit #:** 1

SCID/PSYCHIATRIC HISTORY (continued)

21. Have you ever been prescribed other psychiatric medications (antidepressant, mood stabilizer)		Yes	
		No	
22. How many times have you ever been hospitalized for a psychiatric disorder?	Total number of previous hospitalizations, including current hospitalization in your lifetime:	0	
		1	
		2	
		3	
		≥4	
23. Have you ever been treated as an outpatient or hospitalized for drugs or alcohol problems?		Yes	
		No	
	If yes: How old were you when you were first treated? (years)		
