# Supplementary material for: Environmental and Genetic Contributors to Salivary Testosterone Levels in Infants
Source: Front Endocrinol (Lausanne). 2014 Oct 30;5:187. doi: 10.3389/fendo.2014.00187 (PMC4214198; doi:10.3389/fendo.2014.00187)
Supplement: Supplementary file 1 [file Presentation_1.ZIP › Paternal Psych History.PDF]

Mother Initials: | | | | **Visit Date (mm dd, yyyy):** | | | |

Subject #: | | | | | — | | **Baby #:** | | **Visit #:** | **1** |

PSYCHIATRIC HISTORY – FATHER

1.	Schizophrenia	0.No/unknown	1.Yes	Current __	Past__
2.	Psychotic d/o NOS	0.No/unknown	1.Yes	Current __	Past__
3.	Schizoaffective d/o	0.No/unknown	1.Yes	Current __	Past__
4.	MDD w/ Psychosis	0.No/unknown	1.Yes	Current __	Past__
5.	BPD w/ Psychosis	0.No/unknown	1.Yes	Current __	Past__
6.	Anxiety d/o	0.No/unknown	1.Yes	Current __	Past__
7.	Substance Abuse	0.No/unknown	1.Yes	Current __	Past__
8.	Alcohol Abuse	0.No/unknown	1.Yes	Current __	Past__
9.	Other Psychiatric d/o<br>Specify: ________________________	0.No/unknown	1.Yes	Current __	Past__
10.	Neurodevelopmental d/o<br>Specify: ________________________	0.No/unknown	1.Yes		
11.	Autism	0.No/unknown	1.Yes		

MATERNAL FAMILY PSYCHIATRIC HISTORY (1º Relatives)

1.	Schizophrenia	0.No/unknown	1.Yes	Current __	Past__
2.	Psychotic NOS	0.No/unknown	1.Yes	Current __	Past__
3.	Schizoaffective	0.No/unknown	1.Yes	Current __	Past__
4.	MDD	0.No/unknown	1.Yes	Current __	Past__
5.	BPD	0.No/unknown	1.Yes	Current __	Past__
6.	Anxiety	0.No/unknown	1.Yes	Current __	Past__
7.	Substance Abuse	0.No/unknown	1.Yes	Current __	Past__
8.	Alcohol Abuse	0.No/unknown	1.Yes	Current __	Past__
9.	Other Psychiatric<br>Specify: ________________________	0.No/unknown	1.Yes	Current __	Past__
