# Supplementary material for: Environmental and Genetic Contributors to Salivary Testosterone Levels in Infants
Source: Front Endocrinol (Lausanne). 2014 Oct 30;5:187. doi: 10.3389/fendo.2014.00187 (PMC4214198; doi:10.3389/fendo.2014.00187)
Supplement: Supplementary file 1 [file Presentation_1.ZIP › Pregnancy Summary V1.PDF]

**Mother Initials:**				**Visit Date (mm, dd, yyyy):**				
**Baby Initials:**								
**Subject #:**					—		**Baby #:**		**Visit #:**	**3**

FOCUS PREGNANCY SUMMARY

1. PARITY: Gravidity # ___ ___

2. PARITY: Term Delivery # ___ ___

3. PARITY: Preterm Delivery # ___ ___

4. PARITY: Abortions # ___ ___

5. PARITY: Living Children # ___ ___

6. Number of Fetuses (This Pregnancy) 1 2 3 4 5

7. Premature Labor 0=No/Unknown 1=Yes

8. Preterm Premature Rupture of Membranes (This Pregnancy) 0=No/Unknown 1= Yes

9. Pre eclampsia
   - Pregn. Hypertension 0=No/Unknown 1=Yes Highest BP Systolic: ___ ___ ___
     Highest BP Diastolic: ___ ___ ___
   - Edema 0=No/Unknown 1=Yes
   - Protein uria 0=No/Unknown 1=Yes (If Yes, circle: Trace, 1+, 2+, 3+, 4+)
   - Vision Problems
     - Blurred 0=No/Unknown 1=Yes
     - Scotomata 0=No/Unknown 1=Yes

10. Diabetes (Perinatal) 0=No/Unknown 1=Yes (If yes, circle Type: A1, A2, B, C, Other___)

11. Bleeding 0=No/Unknown 1=Yes Which Trimester: 1 2 3

12. Placental Problems:
    - Previa ___ 0=No/Unknown 1=Yes
    - Abruption___ 0=No/Unknown 1=Yes

13. Polyhydramnios 0=No/Unknown 1=Yes AFI____ (>25)

14. Oligohydramnios 0=No/Unknown 1=Yes AFI____ (< 5)

15. Size for Gestational Age 0=Normal 1=Small (<10%) 2=Large (>90%)

16. Rubella Immune 0=No/Unknown 1=Yes

**Mother Initials:**				**Visit Date (mm, dd, yyyy):**						
**Baby Initials:**										
**Subject #:**					—		**Baby #:**		**Visit #:**	3

17. RH Incompatible 0=No/Unknown 1=Yes Rhogam Given: Yes No (Circle)

18. Other Pregn. Problems 0=No/Unknown 1=Yes
Specify ______________________________, ______________________________
Specify ______________________________, ______________________________

19. Maternal Medical Problems
Specify______________________________, ______________________________

Prenatal Tests

20.	AFP (maternal serum)	0= No/Unknown	1= Normal	2=Abnormal	Value_____	High / Low (Circle
	Estriol	0= No/Unknown	1= Normal	2=Abnormal	Value_____	High / Low (Circle)
	Beta HCG	0= No/Unknown	1= Normal	2=Abnormal	Value_____	High / Low (Circle
	Inhibin	0= No/Unknown	1= Normal	2=Abnormal	Value_____	High / Low (Circle

21. Increased Risk for Down's 0=No/Unknown 1=Yes
Incr. Risk for Trisomy 18 0=No/Unknown 1=Yes
Increased Risk for NTD 0=No/Unknown 1=Yes

Delivery

22. Onset of Labor 0=No Labor 1=Spontaneous 2=Failure to Progress 3=Induced

22.a Maternal Trauma
Lacerations: Yes ___ No ___
Specify Type: Unknown ___
Perineal ___
Vaginal ___

22b. Labor and Delivery Times
Onset of Labor __/___/___ @ ___:___ A P
Membranes Rupt. ___/___/___@___:___ A P
Delivery ___/ ___/___@___:___A P

23. Method of Delivery 0=Vaginal 1=CS If CS: Planned or Emergent (Circle)

24. Forceps 0=No/Unknown 1=Yes

25. Vacuum 0=No/Unknown 1=Yes

26. Breech Position 0=No/Unknown 1=Yes

**Mother Initials:**				**Visit Date (mm, dd, yyyy):**					
**Baby Initials:**									
**Subject #:**					—		**Baby #:**		**Visit #:** 3

Fetal Distress Signs

27. Meconium in Fluid 0=No/Unknown 1=Yes

28. Abnormal EFM 0=No/Unknown 1=Yes
Lates, Variables, Decreased BTB

29. Chorioamnionitis 0=No/Unknown 1=Yes
Antibiotic (this infection)? 0=No/Unknown 1=Yes
Max. maternal temp. ____

Neonate

30. Date of Birth Child ___ ___ / ___ ___ / ___ ___

31. Gender 1. M 2. F

32. Gestational Age at Birth ___ ___ Weeks ___Days

33. Birth Weight (gr.) ___ ___ ___ ___ Range (Circle): <10% 10-24.9% 25-49.9% 50–74.9% 75–89.9% =>90%

34. Left blank intentionally

35. Birth Head Circ. (cm) ___ ___ . ___ Range (Circle): <10% 10-24.9% 25-49.9% 50–74.9% 75–89.9% =>90%

36. Birth Length (cm) ___ ___ . ___

37. APGAR1” ___ ___

38. APGAR5” ___ ___

39. Blood Gasses: PH (arterial) ___ . ___ ___

40. Blood Gasses: PO2 (arterial) ___ ___ . ___

41. Blood Gasses: PCO2(arterial)___ ___ . ___

42. Blood Gasses: BE (arterial) ___ ___ . ___

26.a Neonate Trauma
Cephalohematoma Yes ___ No ___
Foreceps Bruising Yes ___ No ___
Type Unknown ___

Perinatal Complications

43. Nuchal Cord 0=No/Unknown 1=Yes

44. Meconium Aspiration 0=No/Unknown 1=Yes

**Mother Initials:**				**Visit Date (mm, dd, yyyy):**					
**Baby Initials:**									
**Subject #:**					—		**Baby #:**		**Visit #:** 3

---

45. Asphyxia 0=No/Unknown 1=Yes (Use Clinical Description)

46. RH Disease 0=No/Unknown 1=Yes

47. Other Perinatal Problems 0=No/Unknown 1=Yes

Specify: ________________________, ________________________

Postnatal

48. Duration of Stay in Hospital ___ ___ (days)

49. Duration of Stay in NICU ___ ___ (days)

50. Duration of Oxygen ___ ___ (days)

51. Duration of Intubation ___ ___ (days)

52. Jaundice 0=No/Unknown 1=Yes

53. Seizures 0=No/Unknown 1=Yes

54. Sepsis 0=No/Unknown 1=Yes

55. Pneumonia 0=No/Unknown 1=Yes

56. Necrotyzing Enterocolitis 0=No/Unknown 1=Yes

57. Resp. Distress Syndrome 0=No/Unknown 1=Yes

58. Other Postnatal Problems. 0=No/Unknown 1=Yes

Specify: ____

59. Clinical Scan 0=No/Unknown 1=Yes (If Yes, Complete Neonatal U/S Summary)
