# Supplementary material for: Environmental and Genetic Contributors to Salivary Testosterone Levels in Infants
Source: Front Endocrinol (Lausanne). 2014 Oct 30;5:187. doi: 10.3389/fendo.2014.00187 (PMC4214198; doi:10.3389/fendo.2014.00187)
Supplement: Supplementary file 1 [file Presentation_1.ZIP › Pregnancy Summary V2.PDF]

Mother Initials:				Visit Date (mm, dd, yyyy):						
Baby Initials:										
Subject #:	T					—		Baby #:		Visit #: 3

FOCUS PREGNANCY SUMMARY

1. PARITY: Gravidity # ___ ___
2. PARITY: Term Delivery # ___ ___
3. PARITY: Preterm Delivery # ___ ___
4. PARITY: Abortions # ___ ___
5. PARITY: Living Children # ___ ___

6.a. Maternal Weight Gain
Was maternal weight recorded within 2 weeks of delivery? Y / N (circle one)

Weight: ________ lbs.
Date: __ / __ / __

6. Number of Fetuses (This Pregnancy) 1 2 3 4 5
7. Premature Labor 0=No/Unknown 1=Yes (If labor begins <36 wks)
8. Preterm Premature Rupture of Membranes (This Pregnancy) 0=No/Unknown 1= Yes
9. Umbilical Cord Prolapse 0=No/Unknown 1=Yes
10. Pre-eclampsia
    - Pregn. Hypertension 0=No/Unknown 1=Yes Highest BP Systolic: ___ ___ ___
      Highest BP Diastolic: ___ ___ ___
    - Edema 0=No/Unknown 1=Yes
    - Protein uria 0=No/Unknown 1=Yes (If Yes, circle: Trace, 1+, 2+, 3+, 4+)
    - Vision Problems:
      - Blurred 0=No/Unknown 1=Yes
      - Scotomata 0=No/Unknown 1=Yes
11. Diabetes ~~(Perinatal)~~ 0=No/Unknown 1=Yes Other___ (Gestational) ~~(If yes, circle Type: A1, A2, B, C,~~
12. Bleeding 0=No/Unknown 1=Yes Which Trimester: 1 2 3
13. Placental Problems:
    - Previa ___ 0=No/Unknown 1=Yes Total vs Partia (Circle)
    - Abruption___ 0=No/Unknown 1=Yes
14. Rubella Immune 0=No/Unknown 1=Yes
15. RH Incompatible 0=No/Unknown 1=Yes Rhogam Given? Yes No (Circle)
16. Other Pregn. Problems 0=No/Unknown 1=Yes (Ex: Group B Strep. +)
    - Other ______________________________, ______________________________
    - Other ______________________________, ______________________________

16a. Polyhydramnios 0=No/Unknown 1=Yes AFI____ (>25)

16b. Oligohydramnios 0=No/Unknown 1=Yes AFI____ (< 5)

**Mother Initials:**				**Visit Date (mm, dd, yyyy):**							
**Baby Initials:**											
**Subject #:**	T					—		**Baby #:**		**Visit #:**	3

17. Maternal Medical Problems (prior to pregnancy – from Medical History. Ex: HTN)

Specify____________________________, ____________________________

Specify____________________________, ____________________________

18. Was Pregnancy Medically Assisted? (Check all that apply)

a. Fertility Drugs	0=No/Unknown	1=Yes
b. Artificial Insemination	0=No/Unknown	1=Yes
c. In-Vitro Fertilization	0=No/Unknown	1=Yes
d. Reduction	0=No/Unknown	1=Yes
e. Egg Donor	0=No/Unknown	1=Yes
f. Sperm Donor	0=No/Unknown	1=Yes

Prenatal Tests

19.	AFP	0=Unknown	0=Normal	1=Abnormal	Value_____	High / Low (Circle)
	Estriol	0=Unknown	0=Normal	1=Abnormal	Value_____	High / Low (Circle)
	Beta HCG	0=Unknown	0=Normal	1=Abnormal	Value_____	High / Low (Circle)
	Inhibin	0=Unknown	0=Normal	1=Abnormal	Value_____	High / Low (Circle)

20.	Increased Risk for Down's	0=No/Unknown	1=Yes
	Incr. Risk for Trisomy 18	0=No/Unknown	1=Yes
	Increased Risk for NTD	0=No/Unknown	1=Yes

21.	a. Amniocentesis Results:		
	(1). Twin Fetal Lung Maturity	0=No/Unknown	1=Yes
	(2). Chromosomes	0=No/Unknown	1=Yes
	b. CVS Results:	0=No/Unknown	1=Yes
	c. FIRST Results:	0=No/Unknown	1=Yes (If Yes, Positive___, Negative___)

21.d. Onset of Labor: 0=No Labor 1=Spontaneous 2=Failure to Progress and/or Induced (Circle below)
Failure to Progress: 0=No/Unknown, 1=Yes

21.e. Labor and Delivery: Onset of Labor (Date/Time): __/___/___ @ ___:___ A P

21.f. Maternal Trauma Yes ___ No ___
(Perineal or Vaginal Lacerations)
Specify Type: Unknown: _____
Perineal _____
Vaginal _____

21.g. Placental Status (from Pathology Report):
Chorionicity: □ Mono □ Di □ Unknown
Amnionicity: □ Mono □ Di □ Unknown

**Mother Initials:**				**Visit Date (mm, dd, yyyy):**						
**Baby Initials:**										
**Subject #:**	T					—		**Baby #:**		**Visit #:** 3

Delivery – Twin 1

22. Anesthesia 0=No/Unknown 1=Yes
Type:___________________

23. Labor and Delivery Times (Type)
Membranes Rupt. ___/___/___@___:___ A P
Delivery ___/ ___/___@___:___A P

24. Method of Delivery 0=Vaginal 1=C-Section

25. If Vaginal, was Delivery Spontaneous or Operative? (Circle) (Operative = Vacuum or Forceps Used)
Was Vacuum Used? 0=No/Unknown 1=Yes
Were Forceps Used? 0=No/Unknown 1=Yes
Breech Position? 0=No/Unknown 1=Yes

26. If C-Section: Planned or Emergent? (Circle
Primary or Repeat? (Circle)
If Abnormal Presentation, Transverse or Breech? (Circle)

27. If Emergent, Indication for CS: (1) Non-reassuring Fetal Status, (2) Failure to progress,
(Circle all that apply) (3) CPD, (4) Arrest of Descent or Dilation?

27.a. Neonate Trauma
Cephalohematoma Yes ____ No ____
Foreceps Bruising Yes ____ No ____

Fetal Distress Signs – Twin 1

28. Meconium in Fluid 0=No/Unknown 1=Yes

29. Abnormal EFM 0=No/Unknown 1=Yes

30. Chorioamnionitis 0=No/Unknown 1=Yes If Yes, Max. Maternal Temp. _____

**Mother Initials:**				**Visit Date (mm, dd, yyyy):**						
**Baby Initials:**										
**Subject #:**	T					—		**Baby #:**		**Visit #:** 3

Neonate – Twin 1

31. Date of Birth Child ___ ___ / ___ ___ / ___ ___

32. Gender 1. M 2. F

33. Gestational Age at Birth ___ ___ Weeks ___Days

34. Birth Weight (gr.) ___ ___ ___ ___ Range (Circle): <10% 10-24.9% 25-49.9% 50–74.9% 75–89.9% =>90%

35. Birth Head Circ. (cm) ___ ___ . ___ Range (Circle): <10% 10-24.9% 25-49.9% 50–74.9% 75–89.9% =>90

36. Size for Gestational Age 0=Normal 1=Small (<10%) 2=Large (>90%)

37. Birth Length (cm) ___ ___ . ___

38. APGAR1” ___ ___

39. APGAR5” ___ ___

40. Blood Gasses*:* PH (arterial) ___ . ___ ___

41. Blood Gasses*:* PO2 (arterial) ___ ___ . ___

42. Blood Gasses*:* PCO2(arterial)___ ___ . ___

43. Blood Gasses*:* BE (arterial) ___ ___ . ___

Perinatal Complications – Twin 1

44. Nuchal Cord 0=No/Unknown 1=Yes

45. Meconium Aspiration 0=No/Unknown 1=Yes

46. Asphyxia 0=No/Unknown 1=Yes (Use Clinical Description)

46.a. RH Disease 0=No/Unknown 1=Yes

46.b. Other Perinatal Problems 0=No/Unknown 1=Yes

Specify: ____________________________, ____________________________

Specify: ____________________________, ____________________________

Postnatal – Twin 1

47. Duration of Stay in Hospital ___ ___ (days)

48. Duration of Stay in NICU ___ ___ (days)

49. Duration of Oxygen ___ ___ (days)

50. Duration of Intubation ___ ___ (days)

**Mother Initials:**				**Visit Date (mm, dd, yyyy):**			
**Baby Initials:**							

**Subject #:**	**T**					—		**Baby #:**		**Visit #:**	**3**

51.	Jaundice	0=No/Unknown	1=Yes
52.	Seizures	0=No/Unknown	1=Yes
53.	Sepsis	0=No/Unknown	1=Yes
54.	Pneumonia	0=No/Unknown	1=Yes
55.	Necrotyzing Enterocolitis	0=No/Unknown	1=Yes
56.	Resp. Distress Syndrome	0=No/Unknown	1=Yes
57.	Other Postnatal Problems	0=No/Unknown	1=Yes

Specify: ____

Specify: ____

58. Clinical Neonatal U/S Scan Done 0=No/Unknown 1=Yes (If Yes, Complete Summary p. 14)

Mother Initials:				Visit Date (mm, dd, yyyy):							
Baby Initials:											
Subject #:	T					—		Baby #:		Visit #:	3

Delivery – Twin 2

59. Anesthesia 0=No/Unknown 1=Yes
Type:___________________

60. Labor and Delivery Times (Type)
Membranes Rupt. ___/___/___@___:___ A P
Delivery ___/ ___/___@___:___A P

61. Method of Delivery 0=Vaginal 1=C-Section

62. If Vaginal, was Delivery Spontaneous or Operative? (Circle)
Was Vacuum Used? 0=No/Unknown 1=Yes
Were Forceps Used? 0=No/Unknown 1=Yes

63. If C-Section: Planned or Emergent? (Circle
Primary or Repeat? (Circle)
If Abnormal Presentation, Transverse or Breech? (Circle)

64. If Emergent, Indication for CS: (Circle all that apply) (1) Non-reassuring Fetal Status, (2) Failure to progress, (3) CPD, (4) Arrest of Descent or Dilation?

64.a. Neonate Trauma
Cephalohematoma Yes ____ No ____
Foreceps Bruising Yes ____ No ____

Fetal Distress Signs – Twin 2

65. Meconium in Fluid 0=No/Unknown 1=Yes

66. Abnormal EFM 0=No/Unknown 1=Yes

67. Chorioamnionitis 0=No/Unknown 1=Yes If Yes, Max. Maternal Temp. _____

Neonate – Twin 2

68. Date of Birth Child ___ ___ / ___ ___ / ___ ___

69. Gender 1. M 2. F

70. Gestational Age at Birth ___ ___ Weeks ___Days

71. Birth Weight (gr.) ___ ___ ___ ___ Range (Circle): <10% 10-24.9% 25-49.9% 50–74.9% 75–89.9% =>90%

72. Birth Head Circ. (cm) ___ ___ . ___ Range (Circle): <10% 10-24.9% 25-49.9% 50–74.9% 75–89.9% =>90%

73. Size for Gestational Age 0=Normal 1=Small (<10%) 2=Large (>90%)

74. Birth Length (cm) ___ ___ . ___

75. APGAR1" ___ ___

76. APGAR5" ___ ___

77. Blood Gasses: PH (arterial) ___ . ___ ___

78. Blood Gasses: PO2 (arterial) ___ ___ . ___

79. Blood Gasses: PCO2 (arterial) ___ ___ . ___

80. Blood Gasses: BE (arterial) ___ ___ . ___

Perinatal Complications – Twin 2

81. Nuchal Cord 0=No/Unknown 1=Yes

82. Meconium Aspiration 0=No/Unknown 1=Yes

83. Asphyxia 0=No/Unknown 1=Yes (Use Clinical Description)

84. RH Disease 0=No/Unknown 1=Yes

85. Other Perinatal Problems 0=No/Unknown 1=Yes

Specify: __________________________, __________________________

Specify: __________________________, __________________________

Postnatal – Twin 2

86. Duration of Stay in Hospital ___ ___ (days)

87. Duration of Stay in NICU ___ ___ (days)

88. Duration of Oxygen ___ ___ (days)

**Mother Initials:**				**Visit Date (mm, dd, yyyy):**			
**Baby Initials:**							

**Subject #:**	**T**					**—**		**Baby #:**		**Visit #:**	**3**

89. Duration of Intubation ___ ___ (days)
90. Jaundice 0=No/Unknown 1=Yes
91. Seizures 0=No/Unknown 1=Yes
92. Sepsis 0=No/Unknown 1=Yes
93. Pneumonia 0=No/Unknown 1=Yes
94. Necrotyzing Enterocolitis 0=No/Unknown 1=Yes
95. Resp. Distress Syndrome 0=No/Unknown 1=Yes
96. Other Postnatal Problems 0=No/Unknown 1=Yes

    Specify: ____

    Specify: ____
97. Clinical Neonatal U/S Scan Done 0=No/Unknown 1=Yes (If Yes, Complete Summary p.15)
