# Supplementary material for: Environmental and Genetic Contributors to Salivary Testosterone Levels in Infants
Source: Front Endocrinol (Lausanne). 2014 Oct 30;5:187. doi: 10.3389/fendo.2014.00187 (PMC4214198; doi:10.3389/fendo.2014.00187)
Supplement: Supplementary file 1 [file Presentation_1.ZIP › Supplementary Appendices Captions.PDF]

Supplementary Appendices

Maternal Psych History V1: Document for assessment of maternal psychiatric history. Template used for oral interview. This version was used by the study Early Brain Development in High Risk Children. Note that this study includes typically developing singletons in addition to offspring of mothers with psychiatric conditions.

Maternal Psych History V2: Document for assessment of maternal psychiatric history. Template used for oral interview. This version was used by the study Early Brain Development in Twins. Note that this study includes typically developing singletons in addition to offspring of mothers with psychiatric conditions

Paternal Psych History: Document for assessment of paternal psychiatric history. Template used for oral interview. Both studies used this same format.

Maternal Medication History: Document for assessment of maternal medication usage during pregnancy. Template used for oral interview. Both studies used this same format.

Demographic Interview: Document for assessment of demographic information. Template used for oral interview. Both studies used this same format.

Pregnancy Summary V1: Document for assessment of pregnancy history and outcome. Template for medical record review. This version used by the study Early Brain Development in High Risk Children.

Pregnancy Summary V2: Document for assessment of pregnancy history and outcome. Template for medical record review. This version used by the study Early Brain Development in Twins.
